# Memristive and Synaptic Characteristics of Nitride-Based Heterostructures on Si Substrate

**DOI:** 10.3390/nano10050994

**Published:** 2020-05-22

**Authors:** Mehr Khalid Rahmani, Min-Hwi Kim, Fayyaz Hussain, Yawar Abbas, Muhammad Ismail, Kyungho Hong, Chandreswar Mahata, Changhwan Choi, Byung-Gook Park, Sungjun Kim

**Affiliations:** 1 School of Electronics Engineering, Chungbuk National University, Cheongju 28644, Korea; mehrkhalid.2nd@gmail.com (M.K.R.); ismailmalikbzu10@gmail.com (M.I.); chandreswar@gmail.com (C.M.); 2 Inter-University Semiconductor Research Center (ISRC) and the Department of Electrical and Computer Engineering, Seoul National University, Seoul 08826, Korea; minboysky@naver.com (M.-H.K.); rudgh0248@snu.ac.kr (K.H.); 3 Materials Simulation Research Laboratory (MSRL), Department of Physics, Bahauddin Zakariya University Multan Pakistan, Multan 60800, Pakistan; fayyazhussain248@yahoo.com; 4 Department of Physics, Khalifa University, Abu Dhabi 127788, UAE; yawarmju@gmail.com; 5 Division of Materials Science and Engineering, Hanyang University, Seoul 04763, Korea; cchoi@hanyang.ac.kr; 6 Division of Electronics and Electrical Engineering, Dongguk University, Seoul 04620, Korea

**Keywords:** memristor, silicon nitride, boron nitride, neuromorphic computing, resistive switching

## Abstract

Brain-inspired artificial synaptic devices and neurons have the potential for application in future neuromorphic computing as they consume low energy. In this study, the memristive switching characteristics of a nitride-based device with two amorphous layers (SiN/BN) is investigated. We demonstrate the coexistence of filamentary (abrupt) and interface (homogeneous) switching of Ni/SiN/BN/n^++^-Si devices. A better gradual conductance modulation is achieved for interface-type switching as compared with filamentary switching for an artificial synaptic device using appropriate voltage pulse stimulations. The improved classification accuracy for the interface switching (85.6%) is confirmed and compared to the accuracy of the filamentary switching mode (75.1%) by a three-layer neural network (784 × 128 × 10). Furthermore, the spike-timing-dependent plasticity characteristics of the synaptic device are also demonstrated. The results indicate the possibility of achieving an artificial synapse with a bilayer SiN/BN structure.

## 1. Introduction

New memory devices, including phase-change memory (PRAM), spin-transfer torque magnetic memory (STT-MRAM), and resistive switching memory (RRAM), have shown rapid advancement in recent years [1]. PRAM is attaining recognition for 3D Xpoint memory technology due to its reliable operation and exceptional storage class memory (SCM) integration application [2]. STT-MRAM is expanding into various memory markets with rapid latency and non-volatility advantages compared with DRAM. With respect to RRAM, technical immaturity due to variability issues makes it unavailable for certain larger applications. However, RRAM with various resistance change characteristics is still considered as a well-suited option for the use in logic, memory storage, and neuromorphic devices. A significantly large on/off ratio and high endurance are needed for logic applications [3]. The provision of an efficient 3D structure and reliable RRAM operation according to cell size reduction is crucial for high-density memory applications [4]. The utilization of a synaptic device in RRAM requires a multilevel cell (MLC) as well as a low energy operation [5,6]. Gradual set and reset switching is essential to obtain multilevel states; gradual resistive switching occurs using the entire area between the electrode and insulator for interface-type switching [7].

The hardware-based, neuromorphic, analog-type RRAM device plays a synaptic role when externally stimulated by altering its conductance value. The neuromorphic mimicking of human brain activity is capable of processing data in parallel, with greater energy efficiency than that of the existing von Neumann architecture [8,9]. Numerous reports present that metal oxide-based RRAM devices generally have excellent properties [10]. Recently, nitride-based RRAM devices such as AlN, NiN, BN, and SiN also presented exceptional characteristics considering endurance, retention, reliability, and multilevel cell (MLC). Amorphous thin films with abundant traps are considered as a viable option for artificial synaptic material because the conductance can be easily adjusted according to the voltage applied externally [11,12,13,14,15].

In this study, we analyze memristive switching and the synaptic characteristics of two amorphous nitride layers (SiN/BN) on a silicon substrate. For interface-type switching, potentiation and depression are achieved by identical pulse responses to imitate biological learning. Furthermore, biological key synaptic features such as spike-timing-dependent plasticity (STDP) are demonstrated in the Ni/SiN/BN/n^++^-Si structure. Finally, we verified that interface-type switching is significantly suitable for neuromorphic applications by constructing a simple neural network.

## 2. Materials and Methods

The Ni/SiN/BN/Si devices were fabricated as per the following. The n-type dopant (Phosphorus) was implanted into the silicon surface by ion implantations with an acceleration energy of 40 keV. Doses of n^+^ and the n^++^ Si bottom electrode (BE) were 5 × 10^13^ cm^−2^ and 5 × 10^15^ cm^−2^, respectively. Annealing was conducted at 1050 °C for 10 min to restore the damaged silicon lattice during ion-implantation. A 4 nm thick BN was deposited by RF magnetron sputtering using a boron nitride ceramic target on a highly doped silicon substrate at room temperature, and an RF power of 50 W. Before the BN thin-film deposition, the base pressure of the main chamber was maintained at 2 × 10^−6^ torr, and the working pressure was controlled to 4 mTorr by Ar blowing during BN deposition. Subsequently, 4 nm in thickness of SiN was deposited by plasma-enhanced chemical vapor deposition (PECVD) at approximately 300 °C using 5% SiH_4_/N_2_ (800 sccm), NH_3_ (10 sccm), and N_2_ (1200 sccm). Regarding top electrode (TE) deposition, DC magnetron sputtering was used to deposit the 100 nm thick Ni electrodes, with a diameter of 100 μm. All DC voltage sweep electrical properties were developed using a Keithley 4200-SCS and Keysight B1500A semiconductor parameter analyzer. Pulses were measured by the 4225-PMU ultrafast I–V module. For device measurement, the Si bottom electrodes were grounded, and the voltage bias was applied to the Ni-TE.

## 3. Results and Discussion

**Figure 1:**
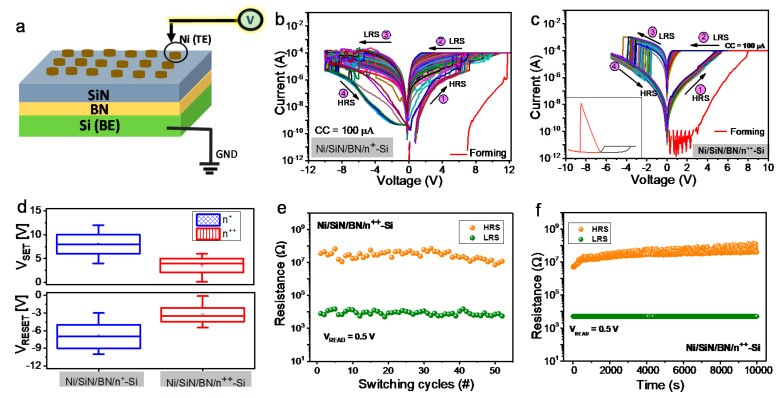
(**a**) Schematic of the Ni/SiN/BN/Si stack and bias configuration. Typical I–V characteristics of (**b**) Ni/SiN/BN/n^+^-Si device and (**c**) Ni/SiN/BN/n^++^-Si device (inset is linear scale for filamentary-like switching); (**d**) Box chart of set and reset voltages of the Ni/SiN/BN/n^+^-Si and Ni/SiN/BN/n^++^-Si devices; (**e**) Endurance; (**f**) Retention of Ni/SiN/BN/n^++^-Si device.

Figure 1a presents the schematic diagram of the Ni/SiN/BN/Si stacked device, and Figure S1a shows the transmission electron microscopy (TEM) image of the double layer device with the Ni/SiN/BN/Si stack. 

The SiN and BN thickness is approximately 4 nm each, making the total thickness of the two dielectrics equal to 8 nm. Both layers are amorphous, thereby making it difficult to accurately distinguish the two; however, approximately 4 nm of each dielectric was deposited during the single-layer deposition of BN, and was confirmed by another TEM image, Figure S1b. 

Next, we investigate the electrical measurement of the fabricated devices. As controlled (reference) devices, a 4 nm thick amorphous SiN device (Ni/SiN/Si) deposited by PECVD is too thin to ensure sufficient switching considering our previous study [13]; and a BN single-layer device (Ni/BN/Si) shows significant variation, as shown in Figure S2. Figure 1b,c shows the current-voltage (I–V) characteristics of the Ni/SiN/BN/n^+^-Si and Ni/SiN/BN/n^++^-Si devices. The device with lightly doped silicon BE (Ni/SiN/BN/n^+^-Si) shows significant variation in switching (i.e., low-resistance state (LRS) and high-resistance state (HRS)) due to the current overshoot during the set process from HRS to LRS [14]. Furthermore, significant switching voltages including set and reset are inevitable due to the series resistance on a low dopant silicon surface, as shown in Figure 1d [15]. Therefore, we focus on the Ni/SiN/BN/n^++^-Si device showing reasonable memristive switching. The typical filamentary-like bipolar resistive switching phenomena under the positive and negative biases are observed in Figure 1c. The device switches from HRS to LRS for the set process and abruptly returns from LRS to HRS for the reset process. Since a tight compliance current (CC, 100 μA) is applied during the set process, the abrupt current jump may not be visible. We verify filamentary-like switching using a higher compliance current (500 μA) to further refine this phenomenon (Figure S3).

**Figure 2:**
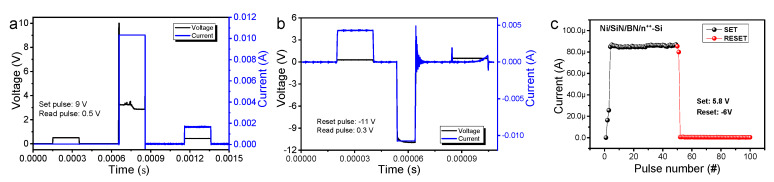
Transient characteristics of (**a**) set pulse of 9 V and (**b**) reset pulse of −11 V. (**c**) Current as a function of 50 consecutive pulse responses (set: 5.8 V and reset: −6 V).

The device is stable over 50 consecutive switching cycles and has a sufficient retention property for 10,000 s, as shown in Figure 1e,f, respectively. To utilize practical operation applications, pulse-driven switching is achieved in the Ni/SiN/BN/n^++^-Si device. Figure 2a shows transient characteristics by the set pulse response (amplitude: 9 V) for the Ni/SiN/BN/n^++^-Si device. The read pulse before and after the set pulse is applied to the device to monitor current. A small amplitude of 0.5 V is used to minimize the read disturbance. After the SET pulse was applied, the current was observed to increase significantly through the read pulse. The reset transient characteristics were similarly observed by applying a negative pulse, Figure 2b. The read pulse of 0.3 V confirms that the current apparently reduced following the reset pulse (amplitude: −11 V). Next, to obtain a multilevel cell (MLC) characteristics, repeated pulses were applied, and the current value was checked for the Ni/SiN/BN/n^++^-Si device, Figure 2c. It is difficult to obtain MLC in filamentary-like switching due to the abrupt current jump. Note, abrupt conductance changes are not suitable for synaptic devices due to difficulty in having multiple synapse weights by pulse signals.

**Figure 3:**
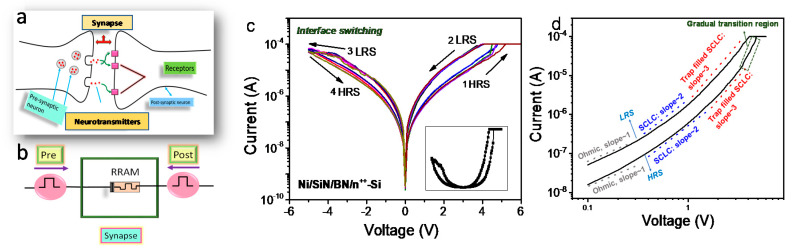
Synaptic characteristics of Ni/SiN/BN/n^++^-Si device: (**a**) schematic of the neuron and synapse network, and (**b**) artificial synaptic memristor. (**c**) Interface switching type I–V curves. (**d**) Log-log fitting curves of the positive region.

Figure 3a displays the biological and artificial neural network schematic where the synapse connects a pre- and postsynaptic neurons.

In the nervous system, a synapse makes a neuron to pass an electrical or chemical signal to another neuron [16,17] by using neurotransmitters. Similarly, a SiN/BN-based memristor can alter its conductance by way of the pre- and postsynaptic neurons (external signals) in Figure 3b. Figure 3c shows the interface-type I–V curves of a Ni/SiN/BN/n^++^-Si device. The device shows the transition from a filamentary to an interface-type switching after approximately 50 switching cycles. To clarify the distinction between filamentary switching and interface switching, we look a bit further at a linear scale (Figure S3). Since the variation of the RRAM device including Ni/SiN/BN/n^++^-Si device is basically large, two switching cases may coexist [18,19]. In the case of this device, the overshoot is large during the initial set operation, and then a high LRS current flows, which leads to an abrupt reset operation. However, as switching was repeated, low LRS current suddenly flowed in a certain cycle with less overshoot, and the switching was switched to interface-type. However, more studies will be needed for the cycling effect.

The fitting process was analyzed to better understand the conduction mechanism of interface-type switching in the Ni/SiN/BN/n^++^-Si device. Figure 3d shows the log-log fitting of an I–V curve in the positive bias. The slope of both, LRS and HRS increases with increasing voltage. There are four distinct regions in the LRS and three distinct regions in the HRS. The first region where the slope is one follows Ohmic conduction. The second region in the LRS and HRS has a slope of 2, following space-charge-limited conduction (SCLC) which is described as follows [20]:

(1) $$J=(9/8)\varepsilon_{r}\varepsilon_{0}\mu (V^{2}/L^{3})\theta_{0}$$

where J is the current density, ε_r_ is the static dielectric constant, ε_0_ is the free space dielectric constant, μ is the electron mobility, V is the applied voltage, L is the dielectric thickness, and *θ*_0_ = (*N_C_*/*Nt*) exp(−*A*/*kT)*, where Nc is the effective density of the states in the conduction band, N_t_ is the density of traps, and the traps are located at energy *A*. The third region with the higher slope (~3) in the LRS and HRS can be explained by the space charge current with the Frenkel effect [21]. The set transition occurs in the fourth region in the HRS with the highest slope (>3). 

**Figure 4:**
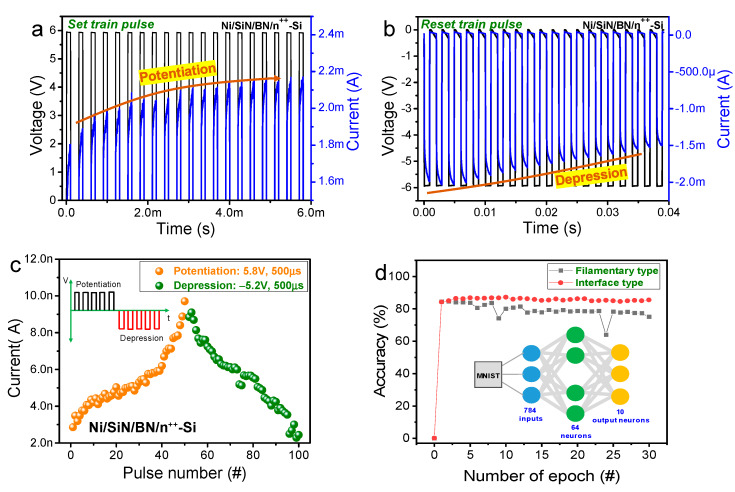
(**a**) Set train pulse and (**b**) reset train pulse of Ni/SiN/BN/n^++^-Si device. (**c**) Long-term potentiation and long-term depression of Ni/SiN/BN/n^++^-Si device. (**d**) Accuracy test in a neural network using Fashion MNIST data set for filamentary and interface-type switching.

To implement additional synaptic characteristics of a Ni/SiN/BN/n^++^-Si device, we analyzed the current change with 20 consecutive identical pulses for a gradual set operation at a fixed voltage (6 V), while gradually decreasing the current with 20 consecutive reset pulses at −6 V, as shown in Figure 4a,b. 

Note that multiple states were well controlled for the synapse array in the hardware-based neuromorphic applications. Furthermore, we strategized for an improved pulse arrangement for long-term potentiation (LTP) and long-term depression (LTD) conductance characteristics by applying repetitive pulses to the device. Following the device voltage application, LTP and LTD characteristics were measured, Figure 4c. To implement these functions, a series of +5.8 V voltage pulses and −5.2 V voltage pulses were applied to the device and the current was measured by a read pulse of 0.5 V after each pulse. The current increases or decreases gradually with the applied pulse number, which is a key operation for hardware-based neuromorphic applications. Figure 4d shows the pattern recognition of the Fashion MNIST classification dataset as a function of epoch for both abrupt and gradual switching modes in a Ni/SiN/BN/n^++^-Si device [21]. The neural network is composed of three layers (784 × 128 × 10) to simulate pattern accuracy. An input image normalization of 28 × 28 pixels is required to keep values between 0 and 1, which is then flattened to a one-dimensional array (784 × 1). A hidden layer of 64 neuron nodes and 10 output neuron nodes corresponds to the 10 different classes of training and test images. Each neural network neuron node is fully connected through memristor devices having quantized weight values that can be updated. We identify varying recognition rates depending on the switching type of a Ni/SiN/BN/n^++^-Si device. An accuracy of 85.6% in the gradual switching mode is better than that of 75.1% in the abrupt switching mode, attributing to larger conductance numbers having better linearity and symmetry in the gradual switching mode. 

**Figure 5:**
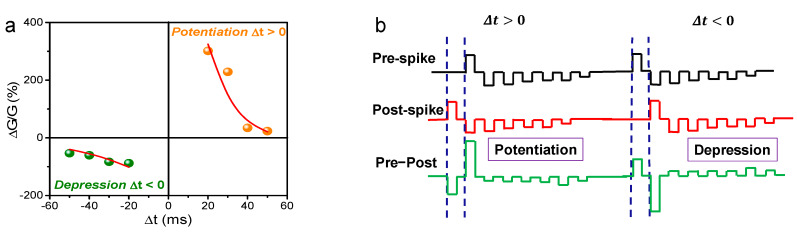
(**a**) Spike-timing-dependent plasticity (STDP)-like curve of Ni/SiN/BN/n^++^-Si device. (**b**) Prespike and postspike pulse scheme to implement STDP.

STDP is the principal synaptic behavior in the Hebbian learning rule which regulates the synaptic weight strength by the time difference between pre- and postspikes [22]. Figure 5a shows STDP-like behavior including potentiation and depression. 

To demonstrate the STDP learning rule, the prespike pulses were applied to the Ni-TE and the postspike pulses were applied to the bottom electrode. The prespike is measured by a train of negative pulses with −7 V, −6.5 V, −6 V, −5.5 V, −5 V, and −4.5 V pulse amplitudes, followed by a train of positive pulses with 7 V, 6.5 V, 6 V, 5.5 V, 5 V, and 4.5 V pulse amplitudes, as shown in Figure 5b. Specific small voltage amplitudes of positive and negative pulses do not affect the conductance, whereas an overlap spiking pulse with a high amplitude causes a conductance change. The conductance of device changes (∆*G*) as a function of ∆t is defined as follows:

(2) $$\Delta G=\frac{G_{final}-G_{initial}}{\min\left(G_{initial},\;\;G_{final}\right)}$$

where *G_initial_* is the initial value of *G* before applying each pair of pulses, *G_final_* is the final *G* after each pulse pair application, and min (*G_i_*, *G_f_*) is the minimum value of *G_initial_* and *G_final_* [23,24]. When a prespike precedes a postspike (Δ*t* > 0), the synaptic weight increase is called set operation, or synaptic potentiation. When a postspike precedes (Δ*t* ˂ 0), the synaptic weight decrease is called reset operation or synaptic depression. The synaptic weight change can be moderated by a time difference. The synaptic weight (∆*w*) function is described as follows:

(3) $$\Delta w=\{\begin{array}{c} A_{+}e^{-\Delta t∕\tau_{+}}\;\;\;\;\;\;\;\;if\;\Delta t>0\\ -A_{-}e^{-\Delta t∕\tau_{-}}\;\;\;\;if\;\Delta t<0 \end{array}$$

where, ∆*w* maximum value is *A*_+_ and *A*_−_ when Δt approaches 0, and *τ*_+_ and *τ*_−_ are the time constants that determine the STDP window temporal spread [25,26].

Synaptic potentiation and depression can be controlled in STDP by spike-timing delay ∆*t*. The STDP features the indication of a conductance change in the memristor as an interval role within the pre- and postspikes. The conductance value increases with decreasing time difference. The shortest spike-timing is applied to the memristor device for potentiation and depression, and the train pulse indicates a significant conductance change. Furthermore, the synaptic weight change contrasted with the spike-timing difference is well-suited for exponential decay functions, indicating that STDP and biological synapse features are comparable [27,28].

## 4. Conclusions

In this study, memristive switching and artificial synaptic characteristics in Ni/SiN/BN/n^++^-Si devices were demonstrated. The gradual set and reset switching achieved was determined to be highly suitable for artificial synapse implementation in a hardware-based neuromorphic. The conduction mechanism of a Ni/SiN/BN/n^++^-Si device was presented to be well-matched with trap-controlled SCLC. The conductance change in a gradual manner was obtained by continuous multiple identical pulses, and superior pattern accuracy in the interface switching mode was observed as compared to the filamentary switching mode. The STDP learning rule was also emulated by systematically applying programmed pre- and postsynaptic spiking pulse trains. 

## Supplementary Materials

The following are available online at https://www.mdpi.com/2079-4991/10/5/994/s1, Figure S1: TEM image of single layer devices. Figure S2: I–V curves of Ni/BN/Si device. Figure S3: Classification of I–V curves in Ni/SiN/BN/n^+^-Si and Ni/SiN/BN/n^++^-Si devices:
