# Supplementary material for: Memristive and Synaptic Characteristics of Nitride-Based Heterostructures on Si Substrate
Source: Nanomaterials (Basel). 2020 May 22;10(5):994. doi: 10.3390/nano10050994 (PMC7279537; doi:10.3390/nano10050994)
Supplement: Supplementary file 1 [file nanomaterials-10-00994-s001.pdf]

Supplementary Materials

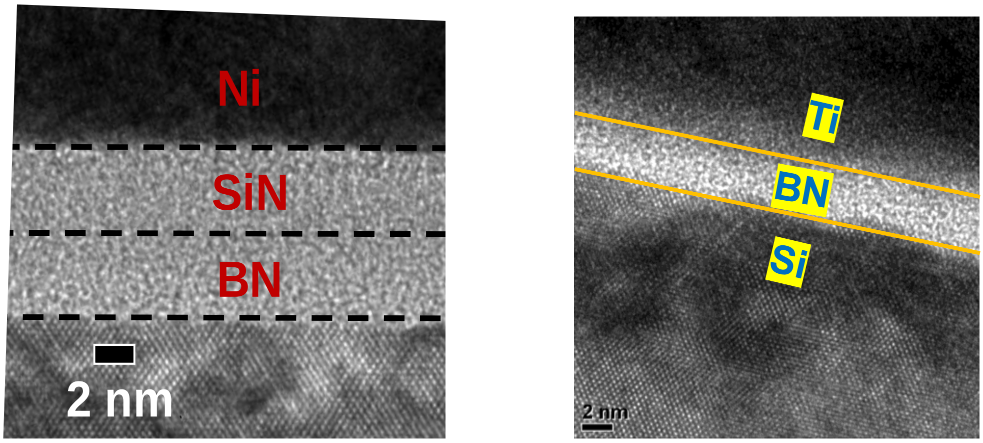

Figure S1. TEM image of (**a**) Ni/SiN/BN/Si device and (**b**) Ti/BN/Si device.

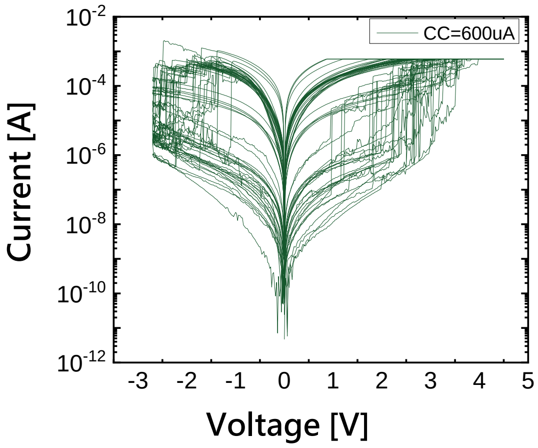

Figure S2. I–V curves of Ni/BN/Si device.

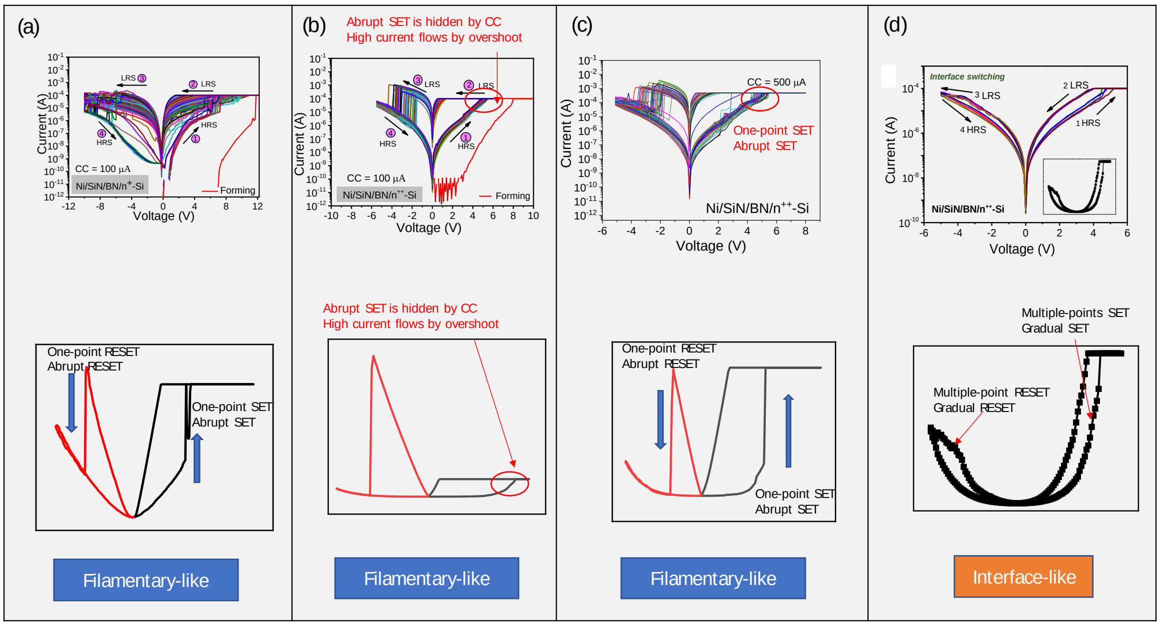

Figure S3. Classification of I–V curves (top: log scale and bottom: linear scale) in Ni/SiN/BN/n+-Si and Ni/SiN/BN/n++-Si devices: (**a**) Filamentary switching in Ni/SiN/BN/n+-Si showing abrupt set and reset with compliance current (CC) of 100 μA. (**b**) Filamentary switching in Ni/SiN/BN/n++-Si with compliance current (CC) of 100 μA. Abrupt set is not clear due to the high current is hidden by CC limitation. The high reset current indicates that high current overshoot occurs during the set operation of 100 μA. (**c**) Filamentary switching in Ni/SiN/BN/n++-Si clearly showing abrupt set and reset with CC of 500 μA. The high CC does not cover the abruptly increasing current. (**d**) Interface switching in Ni/SiN/BN/n++-Si with compliance current (CC) of 100 μA. Gradual set and reset operation are clearly observed in linear scale.
